# Enhanced human receptor binding by H5 haemagglutinins

**DOI:** 10.1016/j.virol.2014.03.008

**Published:** 2014-05

**Authors:** Xiaoli Xiong, Haixia Xiao, Stephen R. Martin, Peter J. Coombs, Junfeng Liu, Patrick J. Collins, Sebastien G. Vachieri, Philip A. Walker, Yi Pu Lin, John W. McCauley, Steven J. Gamblin, John J. Skehel

**Affiliations:** MRC National Institute for Medical Research, The Ridgeway, Mill Hill, London NW7 1AA, UK

**Keywords:** Avian influenza virus, H5N1 influenza virus, Haemagglutinin, Receptor specificity, Receptor binding, Biolayer interferometry, Haemagglutinin crystal structure

## Abstract

Mutant H5N1 influenza viruses have been isolated from humans that have increased human receptor avidity. We have compared the receptor binding properties of these mutants with those of wild-type viruses, and determined the structures of their haemagglutinins in complex with receptor analogues. Mutants from Vietnam bind tighter to human receptor by acquiring basic residues near the receptor binding site. They bind more weakly to avian receptor because they lack specific interactions between Asn-186 and Gln-226. In contrast, a double mutant, Δ133/Ile155Thr, isolated in Egypt has greater avidity for human receptor while retaining wild-type avidity for avian receptor. Despite these increases in human receptor binding, none of the mutants prefers human receptor, unlike aerosol transmissible H5N1 viruses. Nevertheless, mutants with high avidity for both human and avian receptors may be intermediates in the evolution of H5N1 viruses that could infect both humans and poultry.

## Highlights

• H5N1 influenza virus binding.
• Haemagglutinin receptor specificity using biolayer interferometry.
• Haemagglutinin receptor complex crystal structure determination.

## Introduction

Since 1996, highly pathogenic avian influenza viruses (H5N1) have become widespread in poultry and wild birds in Eurasia and Africa and have caused more than 600 zoonotic human infections with a death rate approaching 60% (WHO, 2013). Sporadic human infections continue to occur in countries where H5N1 viruses are endemic, creating a persistent threat to public health and the possibility of virus evolution towards efficient transmission in the human population.

The molecular mechanisms that enable avian influenza viruses to cross the species barrier and transmit efficiently in humans are incompletely understood. However, it has been established for the H1 (1918), H2 (1957) and H3 (1968) pandemic viruses that a change in the binding specificity of the virus membrane glycoprotein, Haemagglutinin (HA), of avian viruses from a preference for α-2,3-linked sialo-saccharides (avian receptor) to a preference for α-2,6-linked sialo-saccharides (human receptor) is a prerequisite for efficient transmission of avian viruses to humans (Connor et al., 1994; Matrosovich et al., 2000). Recent H5 transmission studies also found that a change in the binding preference of the H5 haemagglutinin (HA) from avian receptor to human receptor was necessary to gain aerosol transmissibility among ferrets, the widely accepted animal models for influenza in humans (Chen et al., 2012; Herfst et al., 2012; Imai et al., 2012; Xiong et al., 2013a; Zhang et al., 2013). The receptor binding specificity and transmissibility changes described in all these H5 transmission studies involved the substitution Gln226Leu in HA (in H3 HA numbering here and subsequently), a substitution previously correlated experimentally with the avian to human receptor binding change (Rogers et al., 1983). Furthermore, avian influenza viruses of the H9N2 (Lin et al., 2000) and H7N9 subtypes (Gao et al., 2013; Xiong et al., 2013b) that have also recently caused human infections have HAs with leucine at residue 226. An H5 virus in which the Gln226Leu substitution has occurred has not been isolated from either birds or from humans. However, surveillance of human H5 viruses has revealed a number of H5 mutants that show enhanced human receptor binding with HAs that contain other amino acid substitutions in the receptor binding sub-domain (Chutinimitkul et al., 2010; Gambaryan et al., 2006; Watanabe et al., 2011; Yamada et al., 2006). We have studied the mechanisms by which these mutations alter receptor binding. We have used biolayer interferometry (BLI) to quantitate receptor binding by HAs identified in Southeast Asian clade 1 H5N1 human isolates (Gambaryan et al., 2006; Yamada et al., 2006) and in mutant clade 2.2 viruses that are endemic in Egypt. (Watanabe et al., 2011). The results indicate that two substitutions in H5 clade 1 HA Asn186Lys and Ser227Asn significantly decreased affinity for avian receptor and that the former mutation, which introduces a positive charge, enhanced human receptor binding. The clade 2.2 H5 double mutant Δ133/Ile155Thr HA also had increased affinity for human receptor but had little changed affinity for avian receptor. Based on these results, the human and avian receptor binding modes of H5 HAs bearing the Ser227Asn/Gln196Arg, Asn186Lys, and Δ133/Ile155Thr substitutions were determined by X-ray crystallography. Our observations shed light on the molecular basis of the receptor binding specificity of the mutants and suggest mechanisms for the evolution of HA receptor binding properties during H5 virus infection of humans.

## Results

Recombinant H5N1 viruses were generated by replacing the HA and NA genes of A/Puerto Rico/8/34 (H1N1) (PR8) with corresponding H5N1 HA and NA genes using a previously described reverse genetics system (Hoffmann et al., 2000). cDNA clones of HA genes from A/Vietnam/1194/2004 (H5N1) (VN1194) and A/turkey/Turkey/1/2005 (H5N1) (tyTy) were subcloned into pHW2000 plasmid and their multibasic cleavage sites (QRERRRKKR) were replaced (QRETR). Mutant VN1194 viruses containing HA substitutions – Ser227Asn, Gln196Arg, Asn186Lys, Gly143Arg and selected combinations – Ser227Asn/Gln196Arg and Asn186Lys/Gly143Arg (H3 numbering is used throughout the manuscript unless otherwise stated) and mutant A/turkey/Turkey/1/2005 (H5N1) (tyTy) viruses containing Δ133, Ile155Thr, Ser125Asn and combinations of them were generated by reverse genetics following PCR mutagenesis of their respective wild-type HA cDNAs. All recombinant viruses in this study were propagated and purified from embryonated chicken eggs.

### Receptor binding properties of H5 mutants evaluated by biolayer interferometry (BLI)

**Fig. 1:**
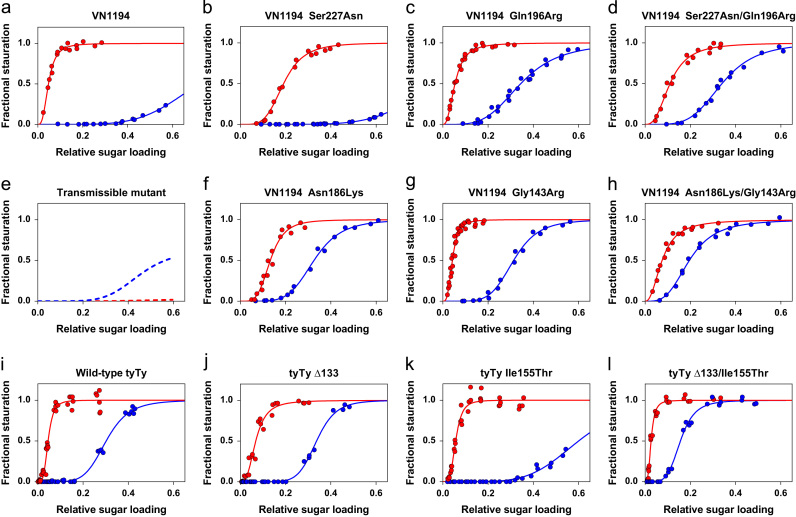
Estimates of the affinity and specificity of receptor binding by mutant H5 influenza viruses. Binding of sialylglycopolymers containing α-2,3-sialolactosamine (3SLN, avian receptor analogue, red) and sialylglycopolymers containing α-2,6-sialolactosamine (6SLN, human receptor analogue, blue) by VN1194 mutants (b–d, f–h) and tyTy mutants (j–l) was characterised by biolayer interferometry (BLI) as detailed before (Lin et al., 2012). For comparison, data for wild-type VN1194 (a) and tyTy (i) viruses and theoretical binding curves for an aerosol transmissible H5 mutant HA (e) (Xiong et al., 2013a) are included.

BLI experiments with mutant clade 1 VN1194 viruses indicated that Ser227Asn (Fig. 1b) and Asn186Lys (Fig. 1f) mutations, located in close proximity to the receptor binding site, reduce avian receptor binding (red curves) by ~1200- and ~80-fold respectively, compared to the wild-type VN1194 (Fig. 1a). For the Ser227Asn mutant, binding to human receptor (blue curves) is weak with an avidity slightly less than wild-type VN1194. In contrast, human receptor binding by the Asn186Lys mutant increases 120-fold. Gln196Arg and Gly143Arg substitutions, located further away from the receptor binding site, modify receptor binding of the VN1194 HA in very similar ways (Fig. 1c and g). Both substitutions in isolation increase virus avidity for human receptor by 120- to 180-fold. However, avidities for avian receptor are almost the same as those of wild-type VN1194. Introduction of the double substitutions Ser227Asn/Gln196Arg and Asn186Lys/Gly143Arg, identified in viruses isolated from humans (Yamada et al., 2006), indicated that the weakened avian receptor binding that resulted from Ser227Asn or Asn186Lys mutations alone can be partially restored by the second substitutions. In the case of the Ser227Asn/Gln196Arg combination, avian receptor binding decreases ~50-fold relative to the VN1194 wild-type as opposed to the ~1200-fold decrease resulting from the Ser227Asn single mutation. For the Asn186Lys/Gly143Arg double mutant, avian receptor binding decreases ~4-fold relative to wild-type in comparison with the ~80-fold decrease in binding by the mutant containing only the Asn186Lys substitution. The second substitutions were also found to increase human receptor binding compared with the Ser227Asn or Asn186Lys single mutants (Fig. 1d and h). The Ser227Asn/Gln196Arg mutant virus has the same avidity towards human receptor as that of the Gln196Arg single mutant, suggesting that increased human receptor binding by the double mutant is mainly brought about by the Gln196Arg substitution (Fig. 1c and d). By contrast the affinity for human receptor of the Asn186Lys/Gly143Arg mutant increases compared with either of the two single mutants; the combined effect gives rise to a ~10^4^-fold gain in human receptor binding avidity compared to the VN1194 wild-type (Fig. 1f, g and h), indicating that both Asn186Lys and Gly143Arg substitutions have a role in improving human receptor binding.

tyTy H5 is the prototype of clade 2.2 viruses that are now established in Egypt. BLI analyses demonstrated that it binds to avian receptor with similar avidity to VN1194. However, it has significantly greater avidity for human receptor (Fig. 1i), which may be partly attributed to the absence of a glycosylation site at 158, previously shown to be important for H5 aerosol transmission (Herfst et al., 2012; Imai et al., 2012). Three mutations, a deletion of residue 133 (Δ133), and two amino acid substitutions, Ile155Thr and Ser125Asn were originally identified as Egyptian strain specific mutations. For HAs containing these mutations BLI analyses indicate that single mutants – Δ133 (Fig. 1j) and Ile155Thr (Fig. 1k), have little effect on avian receptor binding compared to the wild-type tyTy H5 virus (Fig. 1i). Human receptor binding by Δ133 also remains unchanged relative to wild-type tyTy, but there is a ~40-fold decrease in human receptor binding by the Ile155Thr single mutant. Interestingly, when Δ133 and Ile155Thr were introduced simultaneously into tyTy H5, a small increase in avian receptor binding (~3-fold) and a significant increase of human receptor binding (~100-fold) were observed (Fig. 1l). In contrast, the other Egyptian strain specific substitution, Ser125Asn alone or in combination with the Δ133 and/or Ile155Thr mutations has no influence on the receptor binding properties (Supplementary Fig. 1a–d) probably reflecting its relatively distant location from the receptor binding pocket (Supplementary Fig. 2a).

### The structures of the H5 mutant HAs and the complexes that they form with avian and human receptors

BLI analyses indicate that the clade-specific mutations in VN1194 and tyTy HAs have different effects on human and avian receptor binding. The Ser227Asn/Gln196Arg and Asn186Lys/Gly143Arg mutants of VN1194 have increased binding for human receptors but reduced binding for avian receptors. In contrast, the tyTy Δ133/Ile155Thr mutant exhibits increased binding for human receptor with little effect on binding of avian receptor. In order to understand the molecular basis of these differences in receptor binding, crystal structures of VN1194 Ser227Asn/Gln196Arg, Asn186Lys and tyTy Δ133/Ile155Thr mutant HAs in complex with receptor analogues were determined by X-ray crystallography. The crystal structure of an unbound form of the VN1194 Asn186Lys/Gly143Arg mutant was also determined, but the crystal was unsuitable for ligand soaking experiments. Nevertheless, this structure confirms that the Gly143Arg substitution is 15 Å away from the receptor binding site (Supplementary Fig. 2b), and is thus unlikely to affect the binding mode of receptors directly. Its contribution to receptor binding is likely to be electrostatic.

**Fig. 2:**
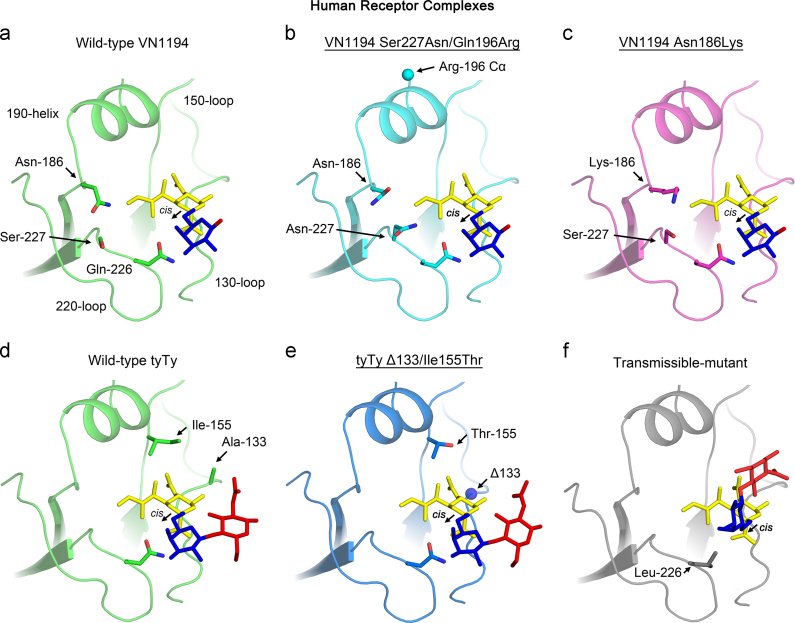
Structures of the receptor binding sites of mutant and wild-type HAs in complex with human receptor analogue as determined by X-ray crystallography. Human receptors bound to HAs of the VN1194 Ser227Asn/Gln196Arg (b), Asn186Lys (c) and tyTy Δ133/Ile155Thr (e) mutants are shown. Four conserved structural elements of the site: 190-helix, 220-loop, 130-loop and 150-loop are in ribbon representation and are labelled. The locations of the amino acid substitutions, together with Gln-226 are indicated. The α-carbon atoms of Arg-196 and the site of deletion, Ala-133 (Δ133, amino-nitrogen of Gly-134), are shown as spheres. The sugar components of the receptor analogues are coloured sialic acid (Sia-1, yellow), galactose (Gal-2, blue) and N-acetylglucosamine (NAG-3, red). Human receptor complexes formed by the Ser227Asn/Gln196Arg (b) and Asn186Lys (c) mutant HAs show electron density for Sia-1 and Gal-2 (Supplementary Fig. 3a and b). The Δ133/Ile155Thr mutant HA–human receptor complex shows good electron density for Sia-1, Gal-2 and NAG-3 (Supplementary Fig. 3c). The directions of the α-2,6-glycosidic bonds are indicated by arrows. Note that all human receptors are bound in *cis*-conformation. For comparison, complexes of human receptors with wild-type VN1194 (a), wild-type tyTy (d), and aerosol transmissible-mutant HAs (f) described before (Xiong et al., 2013a) are shown.

Human receptors bound by the VN1194 Ser227Asn/Gln196Arg (Fig. 2b) and Asn186Lys (Fig. 2c) mutants and the tyTy Δ133/Ile155Thr mutant (Fig. 2e) adopt binding modes similar to those of their respective wild-type HAs (Fig. 2a and d) (Xiong et al., 2013a) (also see Supplementary Fig. 4a–c) and to each other, despite exhibiting significant increases in human receptor binding. The binding mode adopted by human receptors bound to the three mutant HAs is characterised by the face-on orientation (in relation to the view in Fig. 2) of Gal-2, the *cis* glycosidic bond between Sia-1 and Gal-2, and by the receptor exiting the site over the 130-loop. This last characteristic is in marked contrast to the vertical trajectory of exit observed for the human receptor bound to HAs of pandemic H1 (Gamblin et al., 2004; Xu et al., 2012), H2 (Liu et al., 2009) and H3 (Eisen et al., 1997) viruses, and by that of H5 transmissible-mutant (Fig. 2f) (Xiong et al., 2013a).

The human receptor complexes of the two VN1194 mutant HAs also illustrate that the bound receptors are in close proximity to the substitutions Ser227Asn and Asn186Lys, whereas the Gln196Arg substitution is located 17 Å away from the nearest atom of the receptor. Thus, as with the Gly143Arg substitution, the Gln196Arg substitution is also likely to enhance receptor binding electrostatically, consistent with the observation that both substitutions increase the net-charge of HA by +1 and have similar enhancing effects in virus binding assays (compare Fig. 1c and g). In the tyTy Δ133/Ile155Thr human receptor complex, bound receptor is also shown close to the site of the deleted Ala-133 and the Ile155Thr substitution.

**Fig. 3:**
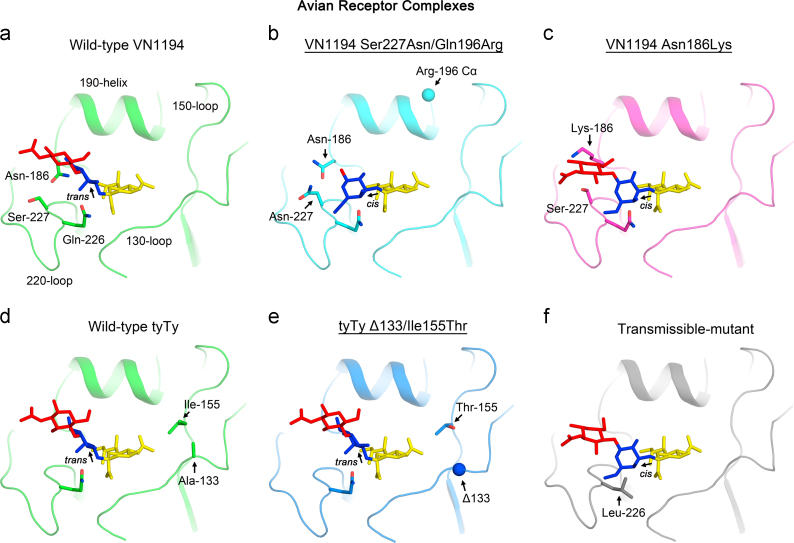
Structures of the receptor binding sites of mutant and wild-type HAs in complex with avian receptor analogue as determined by X-ray crystallography. Avian receptors bound to the VN1194 Ser227Asn/Gln196Arg (b), Asn186Lys (c) and tyTy Δ133/Ile155Thr (e) mutant HAs are shown. The images of the receptor binding sites are rotated through 60° about a vertical axis relative to Fig. 2. The structure of the Ser227Asn/Gln196Arg avian receptor complex shows density for Sia-1 and Gal-2 (Supplementary Fig. 3d), and the avian receptor complexes with Asn186Lys and Δ133/Ile155Thr mutant HAs show electron density for all 3 sugars of the bound receptor (Supplementary Fig. 3e and f). The structural elements and selected residues in the receptor binding sites are indicated as in Fig. 2. For comparison, avian receptors bound to wild-type VN1194 (a), wild-type tyTy (d), and aerosol transmissible-mutant (f) are shown. The directions of the α-2,3-glycosidic bonds are indicated by arrows. Note that the avian receptors bound to the VN1194 Ser227Asn/Gln196Arg mutant (b), the Asn186Lys (c) and the aerosol transmissible-mutant (f) HAs are in *cis*-conformation and the wild-type VN1194 (a) and tyTy (d) HAs and the tyTy Δ133/Ile155Thr mutant (e) HA bind avian receptors in a *trans*-conformation.

The VN1194 mutants – Ser227Asn/Gln196Arg and Asn186Lys also exhibit significant decreases in avian receptor binding in BLI analyses. Their avian receptor complex structures (Fig. 3b and c) show that both mutants bind avian receptor in a fashion similar to that observed for avian receptors bound to the HAs of pandemic viruses and of the H5 aerosol transmissible-mutant (Fig. 3f) (Xiong et al., 2013a) (also see Supplementary Fig. 4d and e). The Sia-1-Gal-2 glycosidic linkages in the two mutant-bound avian receptors are in *cis* rather than the *trans* conformation that is typical for avian receptor bound to wild-type H5 HAs (Fig. 3a and d) (Xiong et al., 2013a). Due to this difference in linkage conformation, Gal-2 in the avian receptors bound by the Ser227Asn/Gln196Arg and Asn186Lys mutants, is rotated ~110° about the 2,3 linked glycosidic bond relative to Gal-2 in the wild-type H5 avian receptor complexes (Fig. 3a and d), making Gal-2 in the mutant bound avian receptor appear face-on (in relation to the view in Fig. 3).

Unlike the VN1194 Ser227Asn/Gln196Arg and Asn186Lys mutants, the tyTy Δ133/Ile155Thr mutant shows a slight increase, rather than loss, in avian receptor binding. Consistent with this observation, avian receptor is found to bind in the same mode (Fig. 3e) as that found in the wild-type tyTy H5 avian receptor complex (Fig. 3d) – a *trans*-glycosidic linkage between Sia-1 and Gal-2 and an edge-on orientation for Gal-2 (in relation to the view in Fig. 3) (also see Supplementary Fig. 4f).

### The importance of interactions between Gln-226 and Asn-186 for avian receptor binding

**Fig. 4:**
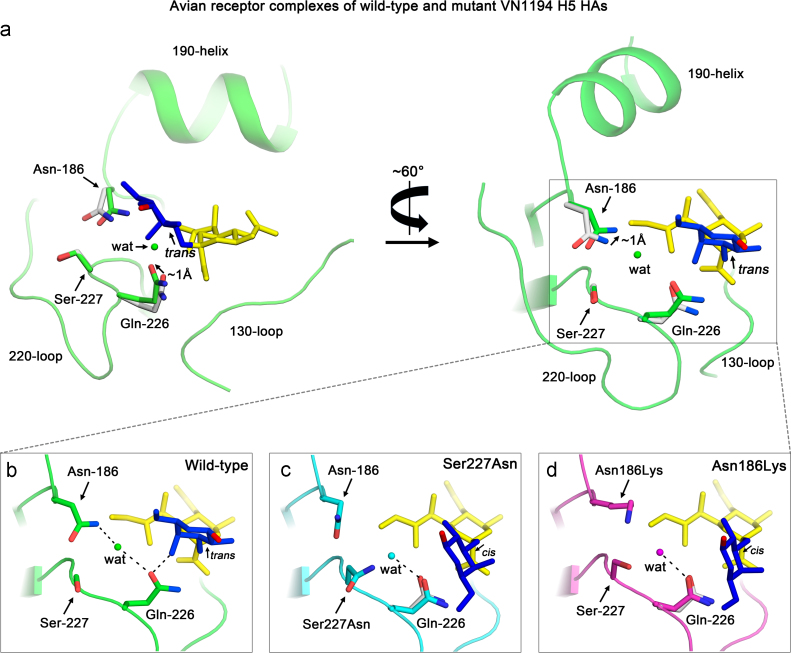
Detailed comparison of wild-type and mutant VN1194 avian receptor complexes. (a) Superposition of unliganded (grey) and avian receptor complexes (green) with wild-type VN1194 in two orientations. The different positions of Gln-226 and Asn-186 upon avian receptor binding are indicated by arrows. (b) A magnified view of the wild-type HA-avian receptor complex shows the water mediated hydrogen bonds between Gln-226, Asn-186, and the avian receptor bound in a *trans* conformation. (c) and (d) Magnified views of the Ser227Asn/Gln196Arg and the Asn186Lys mutant HA complexes with avian receptors. Note that as a result of the point mutation (Asn186Lys) or the change of side-chain rotamer (Ser227Asn), the water mediated hydrogen bond between Gln-226 and Asn-186 is lost, Gln-226 is in a lower position, similar to that of Gln-226 in the un-liganded wild-type HA (shown as grey sticks), and both mutants bind avian receptor in a *cis* conformation.

As described above, the α-2,3-glycosidic bond between Sia-1 and Gal-2 of the avian receptors bound to the Ser227Asn/Gln196Arg (Fig. 3b) and Asn186Lys (Fig. 3c) mutants of VN1194 adopts an unusual *cis* linkage that is usually found in avian receptors bound to HAs derived from pandemic viruses. Structural analyses of the receptor binding sites show that the *trans*- to *cis*-α-2,3-glycosdic linkage switch coincides with loss of a hydrogen bond network that connects Asn-186 to Gln-226 in wild-type HA. When avian receptor is bound to wild-type H5 HA, the side-chain of Gln-226 is ~1 Å higher in the site by comparison with its position in the un-liganded structure (in a structural alignment with an RMSD=0.23 Å on Cα atoms), and forms a hydrogen bond with the 4-hydroxyl of Gal-2 in the receptor (Fig. 4a). The higher position of Gln-226 in the wild-type avian receptor complex appears to be further stabilised by an indirect hydrogen bond between Asn-186 and Gln-226 which is bridged through a conserved water molecule (Fig. 4b). Formation of this hydrogen bond also results in ~1 Å movement of the Asn-186 side-chain by comparison with its position in the un-liganded structure (Fig. 4a). In both the Ser227Asn/Gln196Arg (Fig. 4c) and Asn186Lys (Fig. 4d) mutants, the stabilising hydrogen bond between Asn-186 and Gln-226 is lost, either due to loss of the asparagine side-chain as a result of the Asn186Lys substitution, or to an altered Asn-186 side-chain rotamer influenced by the nearby Ser227Asn substitution. As a consequence, Gln-226 in both mutants adopts a lower position in the site relative to its position in the wild-type avian receptor complex but similar to that in the unliganded wild-type structure (grey sticks in Fig. 4c and d). In this ‘lower’ position it is unable to facilitate binding of avian receptor in the *trans*-conformation, and a distorted binding mode results in which the avian receptor adopts a *cis*-conformation. Although both Ser227Asn and Asn186Lys mutations switch the bound avian receptor to a *cis*-conformation, without changing the human receptor binding mode, they alter receptor affinities to different extents. Compared to the Ser227Asn mutant (Fig. 1b), the Asn186Lys mutant virus has higher avidity for both human and avian receptors (Fig. 1f) achieving similar levels to those observed for the Ser227Asn/Gln196Arg double mutant virus (Fig. 1d). Given that residue 186 does not contact any part of the receptor, it is likely that the Asn186Lys substitution makes an electrostatic contribution to compensate for the loss of avian receptor affinity by addition of positive charge in a similar manner to the Gly143Arg and the Gln196Arg substitutions.

### Enhancement of human and avian receptor binding by the double mutant Δ133/Ile155Thr of clade 2 HA

**Fig. 5:**
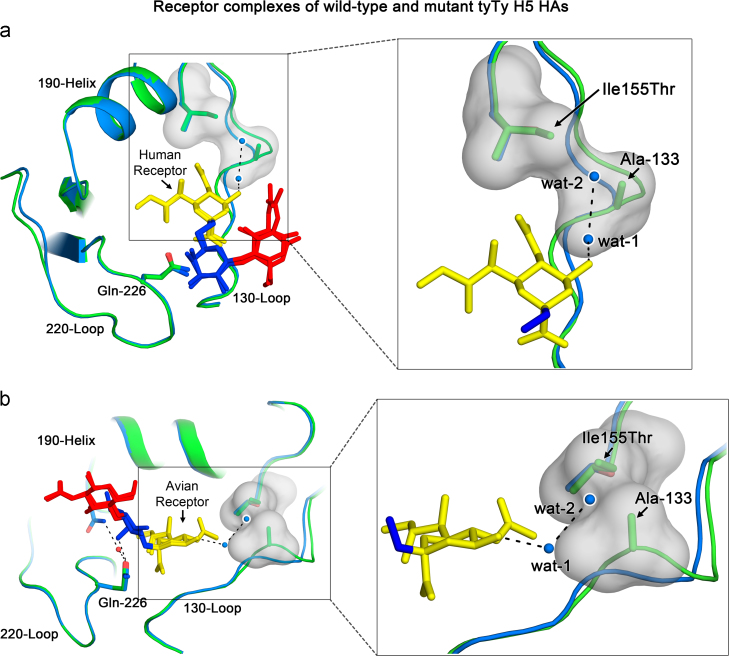
Detailed comparison of wild-type and mutant tyTy receptor complexes. (a) Superposition of the tyTy wild-type (green) and the Δ133/Ile155Thr double mutant (blue) human receptor complexes. The region outlined is magnified on the right side of the figure to show the main-chain structural differences between the wild-type and the double mutant HAs near residue 133. The two water molecules in the receptor complex of the Δ133/Ile155Thr double mutant are shown to be close to the hydrophobic surface (grey) formed by Ile-155 and Ala-133 in wild-type HA. (b) Superposition of the tyTy wild-type (green) and the Δ133/Ile155Thr double mutant (blue) HA-avian receptor complexes. As in (a) the wild-type and the double mutant HAs have differences in main-chain structure near residue 133. The two water molecules in the receptor complex of the Δ133/Ile155Thr double mutant are close to the hydrophobic surface formed by Ile-155 and Ala-133 in wild-type HA.

BLI analyses show that virus bearing the Δ133/Ile155Thr double mutant HA (Fig. 1l) has higher avidity for both avian and human receptors than wild-type HA. Neither mutation alone increases affinity for either human or avian receptor (Fig. 1j and k). Crystallographic analysis of the double mutant reveals that the structure of the sialic acid binding pocket, near residue 133, changes significantly compared to the structure of wild-type tyTy HA. (see Fig. 5a and b). Firstly, the protrusion at residue 133 in the 130-loop is lost as a result of the deletion. Secondly, because of the Ile155Thr substitution, the hydrophobic surface, formed jointly by the side-chain of Ile-155 and the protruding Ala-133 in wild-type HA, is lost. In both the avian and human receptor complexes of Δ133/Ile155Thr mutant, by comparison with the wild-type, two additional water molecules are observed in the vicinity of the mutant residues. They are located near the polar 4-hydroxyl and 5-acetamido groups of bound Sia-1, irrespective of the receptor type (Fig. 5a and b). ‘Wat-1’ forms hydrogen bonds with the 4-hydroxyl of Sia-1, and with ‘Wat-2’. Thus the combination of Δ133 and Ile155Thr mutations creates a more polar environment for the two extra water molecules. The interactions made by these water molecules likely contribute to the higher affinity for both human and avian receptors. Comparison of the wild-type tyTy structure with the structure of the Δ133/Ile155Thr receptor complexes suggests that the presence of either Ala-133 or Ile-155 would restrict the occupancy of these waters due to their close proximity to the water binding sites. Therefore, both substitutions must occur together to allow the water binding necessary for enhanced affinities.

## Discussion

We have carried out structural studies on the HAs of a number of H5 mutants that were isolated from infected humans in Vietnam and Egypt to explain differences in their receptor binding properties. Our results show that mutant VN1194 viruses with HAs containing Ser227Asn or Asn186Lys substitutions, have lower avidity than wild-type virus for avian receptor. This is a result of the loss of a hydrogen-bond network, that in wild-type HA links Gln-226 to Asn-186, and positions Gln-226 about 1 Å higher than in un-liganded HA, to form a hydrogen bond with the 4-OH of Gal-2 of the receptor. Instead, in the mutants, Gln-226 remains lower in the site and the interaction with Gal-2 is not observed. In addition, the α-2,3 glycosidic linkage between Sia-1 and Gal-2, adopts a *cis* configuration in the mutant complex rather than the *trans* conformation that is characteristic of avian HA-avian receptor complexes (Fig. 4). The structural studies with the Δ133/Ile155Thr mutant of tyTy HA reveal a more polar environment near the 130-and 150-loops (Fig. 5), that results from the loss of the hydrophobic surface formed by the side-chains of Ala-133 and Ile-155. These changes correlate with increased avidity for both human and avian receptors.

None of the substituted residues in the mutants we have characterised make direct interactions with the receptors; they appear to have their effects by perturbing the biochemical properties of the receptor binding pocket. Two of the substitutions Gly143Arg and Gln196Arg are distant from bound receptor (see Supplementary Fig. 2b). They appear to increase avidity for receptor electrostatically by the introduction of positive charge. Indeed, together with Asn186Lys, which also increases the net-charge of HA but is closer to bound receptor, the basic substitutions Gly143Arg and Gln196Arg appear to alter the avidity for human and avian receptor to similar extents. It also appears that mutations that increase positive charge or alter site hydration have greater impacts on the more weakly bound receptors. Thus, VN1194 Gln196Arg, Gly143Arg and tyTy Δ133/Ile155Thr gain avidity for human receptor but have little effect on avian receptor binding. These observations can be explained energetically, since the relatively weak effects of these mutations have a greater impact on interactions with low binding energies.

The double mutants – Ser227Asn/Gln196Arg and Asn186Lys/Gly143Arg identified in human isolates of H5 in Southeast Asia (Yamada et al., 2006) both have mutations (Ser227Asn or Asn186Lys) that compromise avian receptor binding. This characteristic may have been selected during human infections to circumvent virus trapping by mucins in the human respiratory tract that are densely decorated with avian receptors. Reduction in avian receptor binding is one of the consequences of mutations observed in the HAs of pandemic viruses that change binding specificity from avian to human receptors. In the HA of the H1 pandemic virus, for example, a Glu190Asp substitution disrupts a hydrogen bond network required for avian receptor binding (Gamblin et al., 2004) and in H2 and H3 pandemic virus HAs, and dramatically in the HA of experimentally transmitted H5 virus, Gln226Leu substitutions also decrease affinity for avian receptors. However, unlike these viruses, none of the H5 mutants analysed here have changed receptor specificity sufficiently to prefer human receptor over avian receptor. The extent of the difference in specificity has been more or less pronounced in different pandemic viruses suggesting that preference itself is the more important characteristic of viruses transmissible in humans. None of the mutations studied here have this characteristic. At the same time retention of an ability to bind avian receptors so that they can infect poultry efficiently is also likely to be important for evolution of a mutant with the potential to transmit in humans. In this regard, our binding and structural data suggest that the Δ133/Ile155Thr mutant is a plausible intermediate since it has the ability to bind avian receptors tightly while at the same time displaying enhanced affinity for human receptor. Indeed surveillance data show that H5 viruses with HAs containing the Δ133/Ile155Thr mutations have been isolated from both poultry and humans in Egypt (Watanabe et al., 2011). Recently also the Δ133/Ile155Thr mutations have been identified in clade 2.1 isolates in Indonesia (Dharmayanti et al., 2011). The widespread distribution of H5 clade 2.2 viruses with Δ133/Ile155Thr mutations many of which also lack glycosylation at residue 158, which is known to be important in aerosol transmission studies in ferrets (Herfst et al., 2012; Imai et al., 2012; Neumann et al., 2012), is a concerning feature of the current stage of evolution of highly pathogenic avian H5 viruses.

## Materials and methods

### Preparation of recombinant viruses

Recombinant H5N1 viruses on A/Puerto Rico/8/34 (H1N1) (PR8) genetic background but containing H5 HA and NA genes, were generated by reverse genetics as previously described (Hoffmann et al., 2000). cDNA clones of HA genes from A/Vietnam/1194/2004 (H5N1) (VN1194) and A/turkey/Turkey/1/2005 (H5N1) (tyTy) were subcloned into pHW2000 plasmid and their multibasic cleavage sites (QRERRRKKR) were replaced (QRETR). By PCR, Ser227Asn, Gln196Arg, Asn186Lys, Gly143Arg in H3 numbering (Ser223Asn, Gln192Arg, Asn182Lys, Gly139Arg in matured H5 HA numbering) and selected combinations Ser227Asn/Gln196Arg and Asn186Lys/Gly143Arg were introduced into the VN1194 HA cDNA. Δ133, Ile155Thr and Ser125Asn (Δ129, Ile151Thr, Ser121Asn mutations in H5 numbering) and combinations of them, were introduced in tyTy HA cDNA. Eight plasmids, each containing one of the 8 viral genes, were co-transfected into co-cultured 293 T and MDCK cells. After 3–5 days, viruses in the supernatants were recovered by passage in MDCK cells and then grown in the allantoic cavity of 10-day old embryonated hen eggs. The candidate vaccine virus NIBRG14, which possesses the HA with modified cleavage site and neuraminidase (NA) from A/Vietnam/1194/2004 and six internal genes of PR8, was obtained from the National Institute for Biological Standards and Control (Wood and Robertson, 2004). Viruses were purified by sucrose gradient sedimentation (Skehel and Schild, 1971). The HA genes of all recombinant viruses were sequenced before and after virus growth in eggs, to confirm their identities.

### Biolayer interferometry

Virus binding to sialic acid receptors was quantitatively evaluated by biolayer interferometry (BLI) on an Octet RED instrument from ForteBio (Menlo Park, CA, USA), as previously described (Lin et al., 2012). Briefly, sialylglycopolymers: α-2,3-sialyllactosamine (3SLN, avian receptor analogue) and α2,6-sialyllactosamine (6SLN, human receptor analogue) linked to a polyacrylamide backbone of 30-kDa polymers containing 20 mol% receptor analogue and 5 mol% biotin were purchased from Lectinity (Moscow, Russia). Sialylglycopolymers were immobilised onto streptavidin biosensors (ForteBio) at 0.01–0.5 μg/ml for ~5 min in 10 mM HEPES, pH 7.4, 150 mM NaCl, 3 mM EDTA, 0.005% Tween-20. Following a buffer wash, biosensors were incubated with virus solution at 100 pM for 30 min to measure the association of virus, in the presence of 10 μM oseltamivir carboxylate (Roche) and 10 μM zanamivir (GlaxoSmithKline) to inhibit the viral neuraminidase. Experiments were performed at 25 °C and sample plates were agitated at 1000 RPM. Responses at equilibrium were normalised with respect to the maximum response for virus binding and plotted as a function of the amount of sugar immobilised on the biosensor, calculated from the response amplitude obtained in the sugar loading step. Experiments were performed at least in duplicate and the data were pooled for fitting. Relative affinities were estimated from apparent *K*_D_*s* calculated from the fractional occupancy as previously described (Xiong et al., 2013a).

### X-ray crystallography

VN1194 HAs containing Ser227Asn/Gln196Arg, Asn186Lys, Asn186Lys/Gly143Arg, and Gln196Arg substitutions, were purified from recombinant viruses grown in chicken eggs as published before (Xiong et al., 2013a). tyTy Δ133/Ile155Thr HA was expressed in Sf-9 insect cells and purified using previously published methods (Lin et al., 2012).

VN1194 Ser227Asn/Gln196Arg, Asn186Lys, and Gln196Arg mutant HAs were crystallised from 0.1 M HEPES/MOPS pH 7.0, 0.05 M MgCl_2_, 28–30% PEG 550 MME using crushed wild-type VN1194 HA crystals as seeds.

VN1194 Asn186Lys/Gly143Arg mutant HA was crystallised from 0.1 M MOPS pH 7.0, 12% PEG 4000.

tyTy Δ133/Ile155Thr mutant HA was initially crystallised from Bis–tris propane pH 7.5, 0.2 M K/NaPO4 (pH 7.0), 20% PEG 3350, and crystals were improved by seeding in Bis–tris propane pH 7.5, 0.05–0.15 M K/NaPO4 (pH 7.0), 15–18% PEG 3350.

Ligand soaking experiments were performed by soaking the crystals in crystallisation solution supplemented with 40 mM receptor analogues for 16 hours. Crystals grown from PEG 4000 and PEG 3350 conditions were cryoprotected with 25% of ethylene glycol prior to freezing.

Diffraction data were collected at 100 K on Diamond beamlines or a Rigaku MicroMax-007 HF X-ray generator coupled to an R-AXIS IV++ detector. Diffraction images were processed in MOSFLM (Leslie and Powell, 2007) or XDS (Kabsch, 2010), before being scaled by Scala (Evans, 2006) in the CCP4 suite (Winn et al., 2011). Structures were determined by molecular replacement in Phaser (McCoy et al., 2007) using the VN1194 structure (PDB:2IBX) (Yamada et al., 2006). Structures were built in Coot (Emsley et al., 2010) and refined in Refmac (Murshudov et al., 1997) before being validated by MolProbity (Chen et al., 2010) to be of good quality. Coordinates (4CQP, 4CQQ, 4CQR, 4CQS, 4CQT, 4CQU, 4CQV, 4CQW, 4CQX, 4CQY, 4CQZ, and 4CR0) are deposited in the PDB and the crystallographic statistics are listed in Supplementary Table 1.
