# Supplementary material for: Enhanced human receptor binding by H5 haemagglutinins
Source: Virology. 2014 May;456-457(100):179–87. doi: 10.1016/j.virol.2014.03.008 (PMC4053833; doi:10.1016/j.virol.2014.03.008)
Supplement: Supplementary file 1 — Supplementary data [file mmc1.pdf]

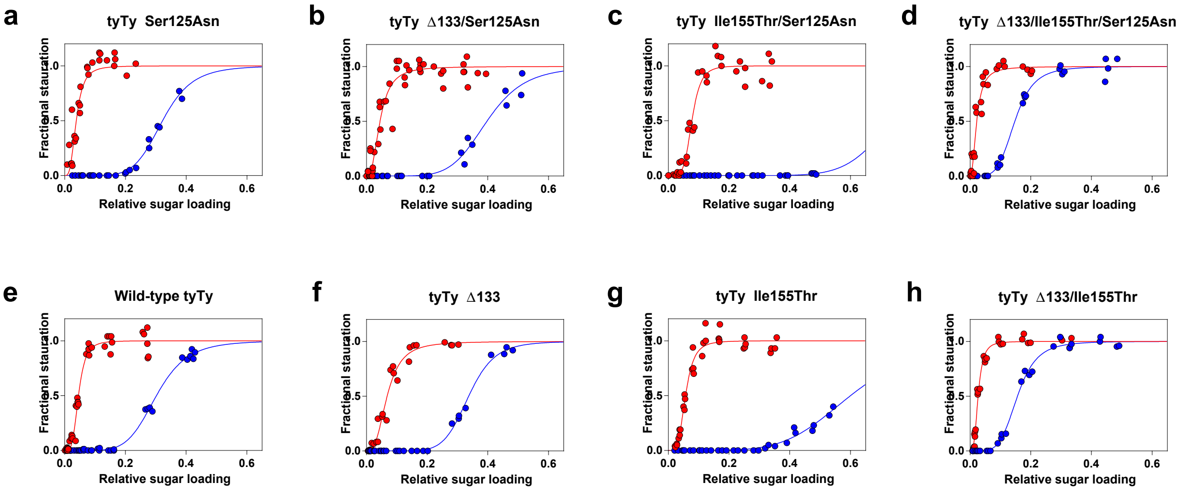

Supplementary Figure 1. The effect of Ser125Asn on receptor binding properties of wild-type and mutant tyTy H5N1 influenza viruses.

Biolayer Interferometry (BLI) binding data of avian (red) and human (blue) receptor analogues by tyTy mutant viruses with either a Ser125Asn single substitution (**a**) or Ser125Asn substitution combined with other amino acid changes (**b-d**). For comparison, data for wild-type tyTy (**e**) and tyTy mutant viruses without the Ser125Asn substitution (**f-h**) are included.

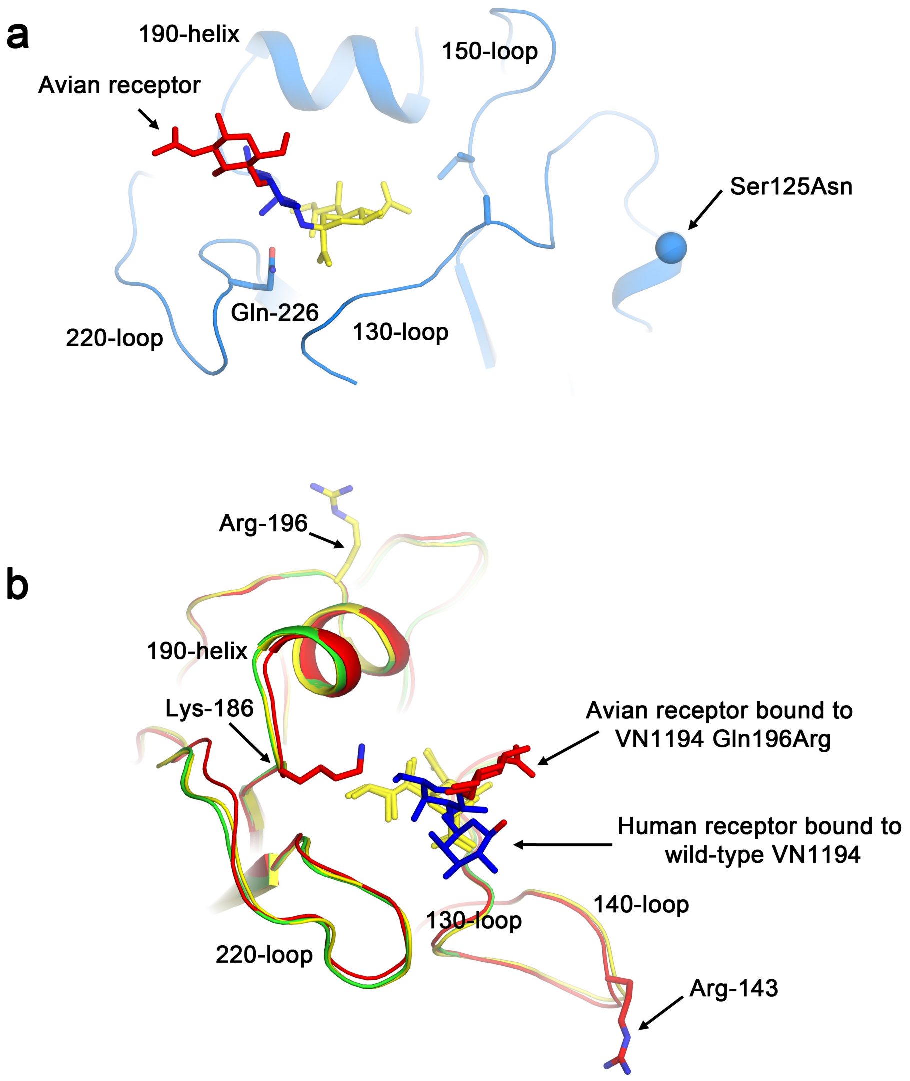

Supplementary Figure 2. Substitutions located further away from the receptor binding pocket.

(**a**), sphere shows the location of Ser125Asn substitution mapped on the receptor binding domain of wild-type tyTy HA. (**b**), overlap of receptor binding domains of VN1194 wild-type (green, in complex with human receptor), Asn186/Gly143Arg mutant (red, unliganded form), and Gln196Arg mutant (yellow, in complex with avian receptor LSTa) shows that substitutions - Gly143Arg and Gln196Arg are far away from the bound receptors.

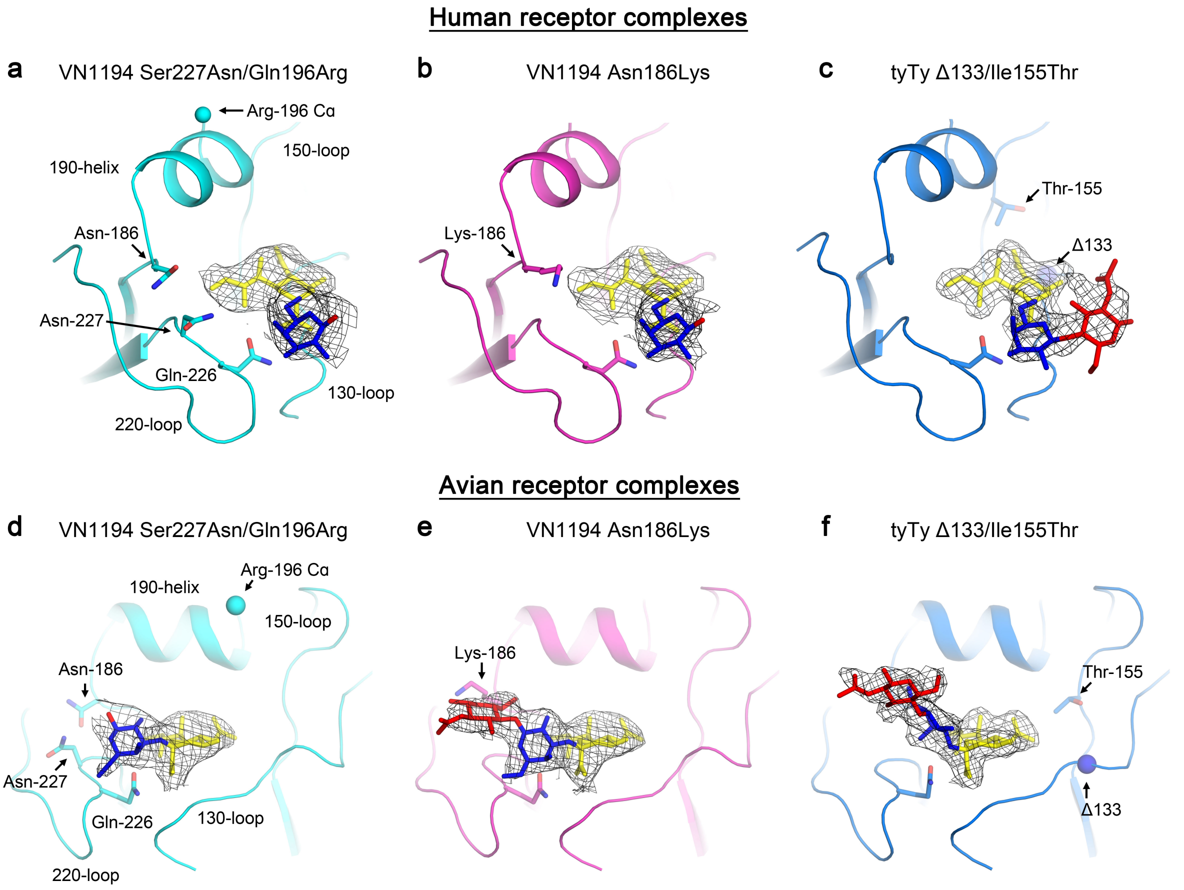

Supplementary Figure 3. Electron density of receptor analogues in complex with mutant H5 HAs.

Electron density (2Fo-Fc, contoured at 0.8σ) of selected receptor analogues bound to receptor binding sites of mutant H5 HAs is shown. Proteins and receptor analogues are coloured and labelled the same as figures in the main text.

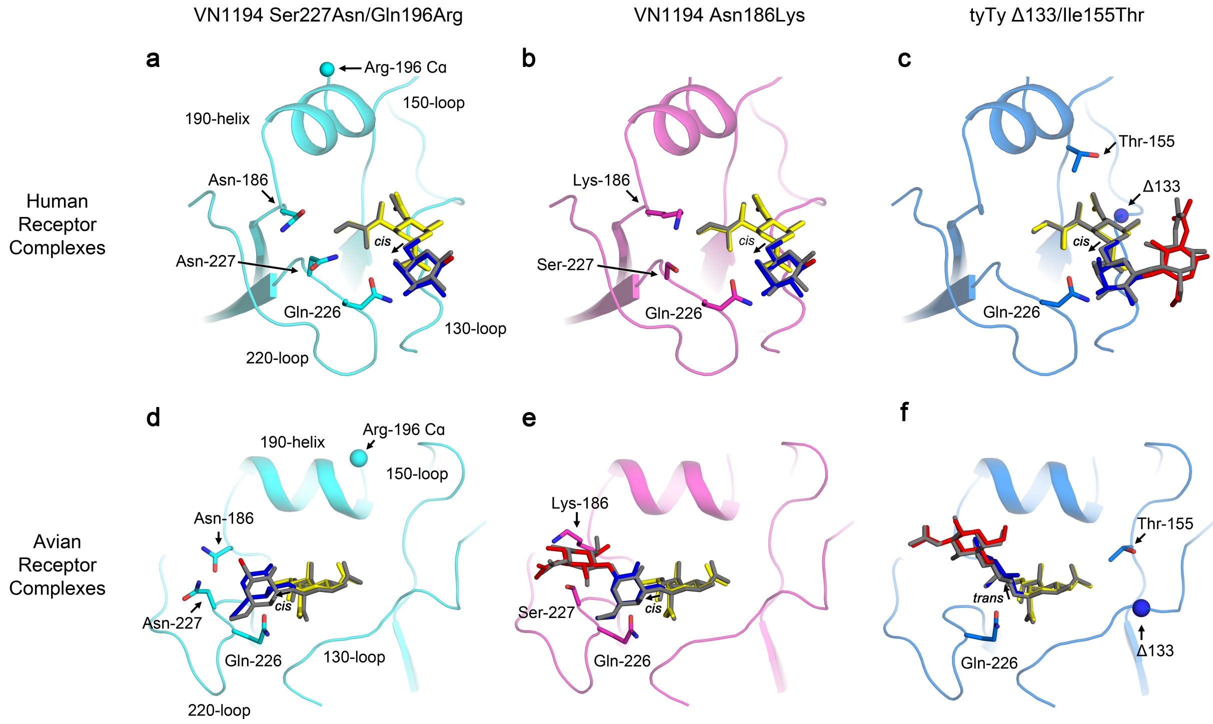

Supplementary Figure 4. Conformations of receptor analogues bound to mutant H5 HAs compared.

(**a-c**), human receptors (coloured) in complex with mutant HAs are compared to human receptors (grey) bound to wild-type HAs. The comparisons show that binding of human receptors by mutant H5 HAs remains almost the same as observed for the wild-types. (**d**) and (**e**), avian receptors (coloured) bound to mutant H5 HAs are compared to that from the H5 transmissible mutant avian receptor complex (grey). (**f**), avian receptor (coloured) bound to tyTy Δ133/Ile155Thr mutant is compared to that from the tyTy wild-type avian receptor complex. The comparisons show that VN1194 mutants - Ser227Asn/Gln196Arg (**d**) and Asn186Lys (**e**) bind avian receptors in a way similar to that observed for the H5 transmissible mutant but distinct from the avian receptor binding mode of the wild-type H5 HAs; binding of avian receptor by tyTy Δ133/Ile155Thr mutant (**f**) remains the same as its wild-type.

Supplementary Table 1. Crystallographic data collection and refinement statistics.

Haemagglutinin	H5 (VN1194) Ser227Asn/Gln196Arg		
**Receptor Analogue**	**APO**	**α2,3SLN**	**α2,6SLN**
**Data collection**			
**X-ray source**	DIAMOND I02	DIAMOND I04-1	DIAMOND I04-1
**Wavelength (Å)**	0.98	0.92	0.92
**Resolution range (Å)**	43.37 - 2.65 (2.79 - 2.65)	37.70 - 2.55 (2.69 - 2.55)	40.81 - 2.45 (2.58 - 2.45)
**Space group**	R32	R32	R32
**a, b, c (Å)**	100.63, 100.63, 448.71	101.67, 101.67, 452.44	101.08, 101.08, 451.34
**α, β, γ (˚)**	90, 90, 120	90, 90, 120	90, 90, 120
**Total reflections**	211680 (29276)	276853 (34796)	174462 (24954)
**Unique reflections**	26059 (2556)	29806 (4179)	33089 (4748)
**Multiplicity**	8.1 (7.9)	9.3 (8.3)	5.3 (5.3)
**Completeness (%)**	100 (100)	99.3 (97.3)	99.5 (99.5)
**Mean I/sigma(I)**	9.7 (2.1)	14.7 (2.6)	10.5 (2.1)
**R-sym (%)**	10.3 (61.9)	8.2 (69.2)	7.4 (61.5)
**Refinement**			
**R-work**	19.33	19.65	20.03
**R-free**	23.79	24.57	23.49
**Protein residues**	495	495	495
**Number of atoms**	4071	4161	4139
**macromolecules**	3866	3863	3866
**receptor Analogue**	-	32	32
**glycans and ligands**	182	169	169
**water**	23	97	72
**RMSD bond length (Å)**	0.006	0.006	0.006
**RMSD bond angle (˚)**	1.070	1.050	1.092
**Ramachandran favored (%)**	97	97	96
**Ramachandran outliers (%)**	0	0	0
**Average B-factor**	101.6	87.5	88.8
**macromolecules**	99	85.9	86.8
**receptor Analogue**	-	106.5	104.4
**glycans and ligands**	161.1	129.9	132.8
**water**	73.3	64.6	68.8
**PDB Code**	4CQP	4CQQ	4CQR

Haemagglutinin		H5 (VN1194) Asn186Lys	
Receptor Analogue	APO	α2,3SLN	α2,6SLN
**Data collection**			
X-ray Source	DIAMOND I04-1	DIAMOND I04-1	DIAMOND I04-1
Wavelength (Å)	0.92	0.92	0.92
Resolution range (Å)	39.43 - 2.55 (2.69 - 2.55)	50.79 - 2.45 (2.58 - 2.45)	35.70 - 2.48 (2.61 - 2.48)
Space group	R32	R32	R32
a, b, c (Å)	101.20, 101.20, 451.67	101.58, 101.58, 452.98	101.46, 101.46, 452.19
α, β, γ (˚)	90, 90, 120	90, 90, 120	90, 90, 120
Total reflections	271552 (36535)	262405 (36141)	305665 (43303)
Unique reflections	29712 (4279)	33498 (4768)	32430 (4655)
Multiplicity	9.1 (8.5)	7.8 (7.6)	9.4 (9.3)
Completeness (%)	99.93 (100)	99.2 (98.1)	100 (100)
Mean I/sigma(I)	16.66 (3.2)	13.1 (2.7)	17.8 (2.8)
R-sym (%)	8.3 (63.0)	8.5 (67.5)	6.8 (68.0)
**Refinement**			
R-work	19.47	20.04	20.42
R-free	23.23	22.66	24.13
Protein residues	495	495	495
Number of atoms	4209	4241	4166
macromolecules	3863	3863	3863
receptor Analogue	-	46	32
glycans and ligands	182	169	169
water	164	163	102
RMSD bond length (Å)	0.006	0.005	0.005
RMSD bond angle (˚)	1.038	1.052	1.030
Ramachandran favored (%)	97	98	97
Ramachandran outliers (%)	0	0	0
Average B-factor	79	78.4	85.1
macromolecules	77.3	76.4	83.3
receptor Analogue	-	106.5	105.1
glycans and ligands	128.2	123.6	129
water	64.9	64.6	66.9
PDB code	4CQS	4CQT	4CQU

Haemagglutinin	H5 (tyTy) Δ133/Ile155Thr			
**Receptor Analogue**	**APO**	**α2,3SLN**	**α2,6SLN**	**LSTa**
**Data collection**				
**X-ray source**	DIAMOND I03	DIAMOND I04-1	DIAMOND I04	DIAMOND I04
**Wavelength (Å)**	0.98	0.92	0.98	0.98
**Resolution range (Å)**	70.03 - 2.86 (3.01 - 2.86)	101.06 - 2.3 (2.42 - 2.3)	87.73 - 2.3 (2.42 - 2.3)	48.69 - 2.05 (2.16 - 2.05)
**Space group**	$P2_1$	$P2_1$	$P2_1$	$P2_1$
**a, b, c (Å)**	87.41, 117.25, 101.00	87.64, 117.55, 101.16	87.82, 117.10, 101.28	89.00, 116.35, 101.84
**α, β, γ (˚)**	90, 92.52, 90	90, 92.5, 90	90, 92.58, 90	90, 91.69, 90
**Total reflections**	141094 (20466)	316019 (46382)	311931 (46024)	443314 (60212)
**Unique reflections**	46268 (6764)	89337 (13055)	90479 (13220)	125504 (18119)
**Multiplicity**	3.0 (3.0)	3.5 (3.6)	3.4 (3.5)	3.5 (3.3)
**Completeness (%)**	98.3 (99.0)	98.3 (98.7)	99.6 (100.0)	96.64 (95.44)
**Mean I/sigma(I)**	8.8 (2.0)	11.9 (2.0)	9.9 (2.0)	8.07 (1.94)
**R-sym (%)**	8.0 (51.7)	8.4 (65.9)	9.0 (64.8)	10.6 (63.3)
**Refinement**				
**R-work**	19.53	19.56	18.57	19.27
**R-free**	24.44	23.79	22.61	22.67
**Protein residues**	1463	1466	1462	1467
**Number of atoms**	11976	12709	12610	13227
**macromolecules**	11610	11636	11562	11638
**receptor Analogue**	-	138	138	138
**glycans and ligands**	142	131	151	136
**water**	224	804	759	1315
**RMSD bond length (Å)**	0.006	0.006	0.007	0.006
**RMSD bond angle (˚)**	1.170	1.071	1.238	1.095
**Ramachandran favored (%)**	97	97	97	98
**Ramachandran outliers (%)**	0	0	0	0
**Average B-factor**	79.4	49.0	51.2	42.0
**macromolecules**	79.8	49.6	51.1	41.7
**receptor Analogue**	-	46.3	71.7	45.1
**glycans and ligands**	95.4	49.5	70.9	48.1
**water**	53.6	40.7	45.4	43.4
**PBD code**	4CQV	4CQW	4CQX	4CQY

Haemagglutinin	H5 (VN1194) Gln196Arg	H5 (VN1194) Asn186Lys/Gly143Arg
Receptor Analogue	LSTa	APO
**Data collection**		
X-ray Source	Rigaku MicroMax-007 HF	DIAMOND I02
Wavelength (Å)	1.54	0.98
Resolution range (Å)	74.65 - 2.70 (2.85 - 2.70)	67.51 - 2.65 (2.79 - 2.65)
Space group	R32	$P6_3$
a, b, c (Å)	101.15 101.15 447.89	77.95, 77.95, 210.411
α, β, γ (˚)	90, 90, 120	90, 90, 120
Total reflections	129854 (11837)	115461 (13157)
Unique reflections	24883 (3565)	20820 (2859)
Multiplicity	5.2 (3.3)	5.5 (4.6)
Completeness (%)	99.9 (99.4)	99.1 (93.7)
Mean I/sigma(I)	11.7 (1.9)	9.8 (2.6)
R-sym (%)	7.7 (43.0)	14.0 (54.9)
**Refinement**		
R-work	21.72	22.45
R-free	25.22	26.2
Protein residues	483	485
Number of atoms	4080	4023
macromolecules	3864	3837
receptor Analogue	46	-
glycans and ligands	83	56
water	87	130
RMSD bond length (Å)	0.005	0.007
RMSD bond angle (˚)	0.982	1.163
Ramachandran favored (%)	97	94
Ramachandran outliers (%)	0	0
Average B-factor	75.9	42
macromolecules	75.3	42.2
receptor Analogue	90.3	-
glycans and ligands	104.3	51.2
water	60.2	29.9
PDB code	4CQZ	4CR0
